# Trends and Outcomes of Alcoholic Acute Pancreatitis in Patients with Alcohol Use Disorder Treated with Naltrexone in the United States: Before and After the COVID-19 Pandemic

**DOI:** 10.1007/s10620-025-09411-2

**Published:** 2025-09-29

**Authors:** Thai Hau Koo, Alexander Malik, Mohammad Zaahid Sheriff, Rishi Chowdhary, Adrian Lindsey

**Affiliations:** 1 Gastrointestinal Function and Motility Unit, Hospital Pakar Universiti Sains Malaysia, Kota Bharu, Kelantan Malaysia; 2 Division of Gastroenterology and Hepatology, MetroHealth Medical Center, Case Western Reserve University, Cleveland, OH USA; 3 Cleveland VA Hospital Systems, Cleveland, OH USA; 4 Department of Medicine-Pediatrics, MetroHealth Medical Center, Cleveland, OH USA; 5 Case Western Reserve University, Cleveland, OH USA

**Keywords:** Alcohol-use disorder, Pancreatitis, Alcoholic, Naltrexone, COVID-19

## Abstract

**Background:**

The role of naltrexone in improving acute alcoholic pancreatitis (AAP) outcomes in alcohol use disorder (AUD) patients, remains unclear.

**Aim:**

We aimed to assess the temporal trends in AAP incidence and the impact of naltrexone use on clinical outcomes in AUD patients.

**Methods:**

We conducted a retrospective cohort study using de-identified patient data from the TriNetX database, including all adults (≥ 18 years) diagnosed with AUD. AAP patients were then identified and stratified by naltrexone exposure. The incidence and prevalence of AAP in AUD patients were assessed pre-(March 2015–2019) and post-COVID-19(March 2020–2025). Propensity score matching (PSM) (1:1) was performed to adjust for confounders. Univariate Cox proportional hazards model was used to assess the outcomes including mortality, chronic pancreatitis, alcoholic hepatitis, cirrhosis, hospital readmission, and emergency department (ED) visits at 1, 6, and 12 months. Standardized mean differences < 0.1 ensured well-matched cohorts.

**Results:**

Among 1,331,338 AUD adults, 33,561 (2.52%) developed AAP. The incidence of AAPs increased significantly following the COVID-19 pandemic, with the largest relative rise among younger adults (18–34 years), women, and racial minorities. Of those with AAP, 1,871(5.6%) received naltrexone. Following PSM, naltrexone use was associated with significantly lower odds of mortality at one (OR 0.274, *p* < 0.0001) and six months (OR 0.571, *p* = 0.005). Reduced odds were also observed for alcoholic hepatitis at one month and hospital readmissions across all time points. Surprisingly, naltrexone use was associated with increased odds of ED visits.

**Conclusions:**

The incidence of AAP has increased since COVID-19 onset. Naltrexone may improve short-term outcomes in patients with AUD and AAP.

**Supplementary Information:**

The online version contains supplementary material available at 10.1007/s10620-025-09411-2.

## Introduction

Acute pancreatitis (AP) is a common reason for gastrointestinal-related hospitalization, with gallstones and alcohol use accounting for approximately 80% of cases [1, 2]. Alcohol use disorder (AUD) markedly increases the risk of alcoholic AP (AAP) and its complications. The COVID-19 pandemic has led to documented increases in alcohol consumption and related harm (e.g., alcohol-induced deaths) [3]. However, data on how these shifts have affected the incidence of AAP are scarce. Meanwhile, medications for AUD (MAUD), such as naltrexone, an opioid antagonist, have proven efficacy in reducing heavy drinking and improving outcomes in AUD patients [4]. For example, large trials and meta-analyses show that oral naltrexone (50 mg daily) reduces rates of return to heavy drinking (number needed to treat ≈11 vs. placebo) and is recommended by Substance Use Guidelines Committee and American Association for the Study of Liver Diseases (AASLD) as first-line pharmacotherapy for AUD [4–6]. Naltrexone has also been associated with better outcomes (e.g., lower decompensation and mortality) in patients with alcohol-associated liver disease (ALD) [7]. Whether naltrexone use influences outcomes in patients with AUD who develop AAP is unknown.

Therefore, we aimed to evaluate national trends in AAP incidence before versus after COVID-19 and to compare clinical outcomes in AUD patients with AAP who did or did not receive naltrexone in a large population-based US cohort.

## Methods

We utilized the U.S. Collaborative Network of TriNetX (with 68 healthcare organizations [HCOs]), an aggregated, de-identified electronic health record platform, to identify adult inpatients (≥ 18 years) with confirmed AUD between March 2015 and March 2025. From this AUD cohort, we selected those who developed concurrent AAP (alcohol was identified as the etiologic factor for AP via diagnostic codes and exclusion of other causes; see Supplement Material). We defined the pre-COVID period as March 2015–February 2020 and post-COVID period as March 2020–March 2025. The incidence and prevalence of AAP in the AUD cohort were compared across these periods (overall, and stratified by age, sex, race, and ethnicity). Among AUD patients with AAP, we created a pairwise matched comparison between those who were prescribed naltrexone during the index AAP hospitalization or within 7 days of discharge, and those who received no-naltrexone. A complete list of exclusion criteria—e.g., other etiologies of pancreatitis, advanced liver disease, or concurrent substance abuse that could confound outcomes—is provided in the Supplemental Material.

We applied 1–1 propensity score matching (PSM) using nearest-neighbor algorithms without replacement. Covariates in the propensity model included demographics, relevant comorbidities, and risk factors for pancreatic and liver outcomes (detailed in Supplemental Material). In brief, we matched on age, sex, race/ethnicity, major comorbid conditions (diabetes, obesity, etc.), baseline liver disease severity markers (prior alcoholic hepatitis, etc.), psychiatric comorbidities (mood, anxiety disorders and others), and indicators of AUD severity (e.g., history of withdrawal management, concurrent use of benzodiazepines or other AUD medications). Balance between matched groups was assessed with standardized mean differences (target < 0.1 for all covariates).

We then evaluated clinical outcomes at 1, 6, and 12 months from the index AAP hospitalization. The primary outcome was all-cause mortality. Secondary outcomes included new diagnoses of alcoholic hepatitis, new or worsening cirrhosis, new chronic pancreatitis, all-cause hospital readmission, and emergency department (ED) visits. Time-to-event analyses were conducted using univariate Cox proportional hazards models on the matched cohorts for each outcome. In addition, we calculated odds ratios (OR) and 95% confidence intervals (CI) for each outcome at each time point using logistic regression on matched data (equivalent to conditional odds within pairs). A two-sided *p* < 0.05 was considered statistically significant. This study followed the Strengthening the Reporting of Observational Studies in Epidemiology (STROBE) reporting guidelines for cohort studies (Supplemental Checklist).

## Results

### AAP Incidence Trends

**Fig. 1:**
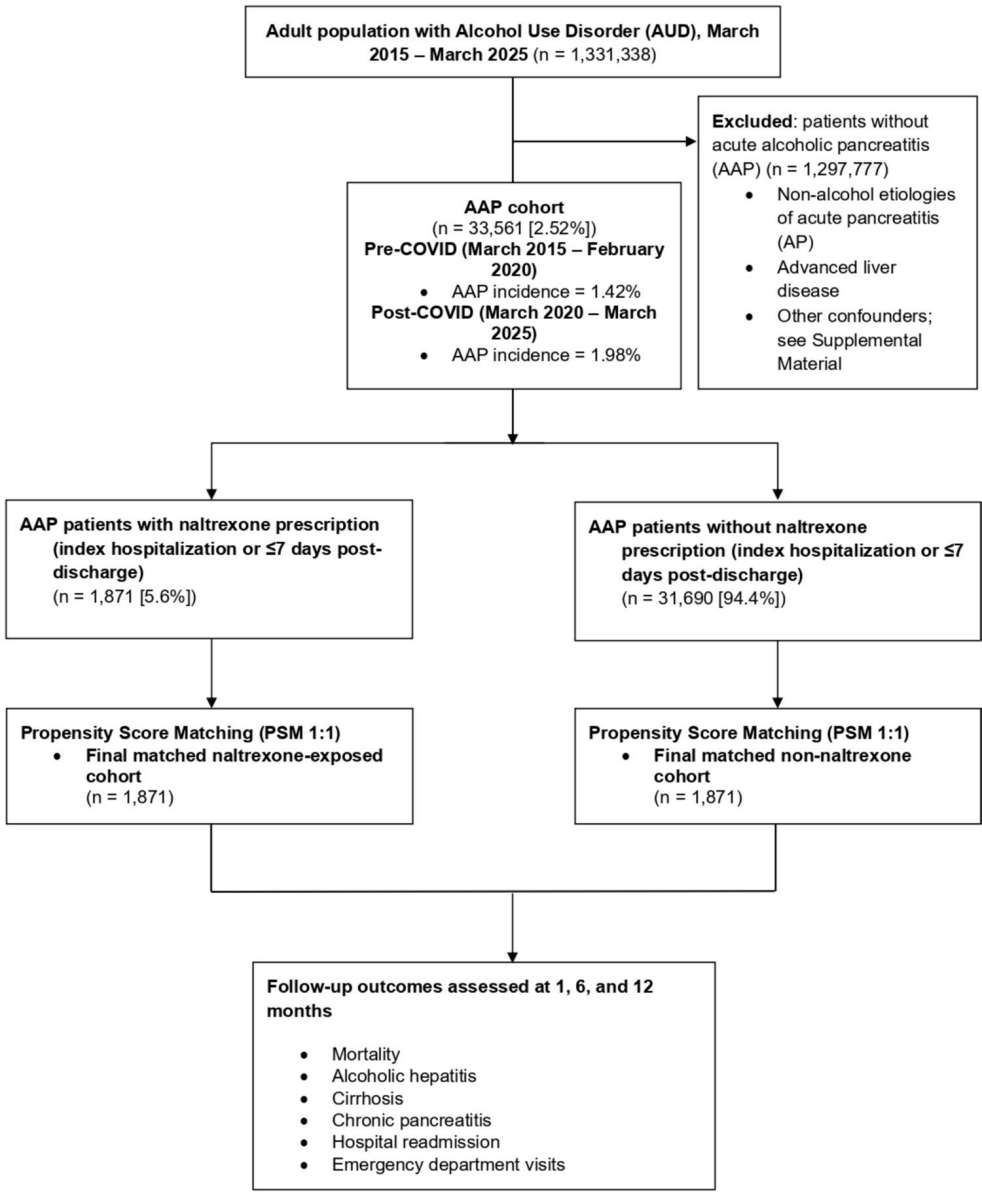
CONSORT diagram of study/control cohorts’ selection flow

Among 1,331,338 patients with AUD in the database, 33,561 (2.52%) experienced at least one episode of AAP during the study period. The incidence of AAP was significantly higher in the post-COVID era compared to pre-COVID (1.98% vs. 1.42%, respectively; Table 1A). This represents a notable relative increase in risk of pancreatitis after 2020. Subgroup analysis revealed that the rise in AAP incidence was most pronounced among younger adults. For example, in the 18–24 year-old age group, the incidence doubled from 1.22% (pre-2020) to 2.22% (post-2020). Similar upward shifts were seen in ages 25–34 (2.11% to 3.33%) and 35–44 (2.41% to 3.74%). Female patients with AUD showed an increase in AAP incidence from 1.21 to 1.78% pre- vs post-pandemic, while male patients increased from 1.52 to 2.09%. All major racial/ethnic groups experienced rises as well—for instance, among Hispanic/Latino patients the incidence rose from 1.33 to 2.15%. Figure 1 provides a visual timeline of AAP incidence over 2015–2024, highlighting the uptick coincident with the pandemic’s onset.

**Table 1:** (A) Incidence of Alcoholic Acute Pancreatitis (AAP) in alcohol use disorder (AUD) Patients (n = 1,331,338) Pre- vs. Post-COVID-19; (B) Baseline characteristics, propensity score matching, and clinical outcomes of AUD Patients with AAP—With vs. Without Naltrexone

(A) Stratum	Pre-COVID (%)	Post-COVID (%)	Interpretation
Overall	1.42	1.98	AAP incidence increased post-COVID
*Age Group (years)*			
18–24	1.22	2.22	Sharpest increase in 18–24 age group
25–34	2.11	3.33	Significant rise in younger adults
35–44	2.41	3.74	Consistent rise in mid-age adults
45–54	2.18	3.21	AAP incidence rising in midlife
55–64	1.20	1.54	AAP persists in older adults
65–74	0.57	0.79	Stable but low incidence in elderly
≥ 75	0.35	0.75	Small n; interpret with caution
*Sex*			
Female	1.21	1.78	Incidence increased in females
Male	1.52	2.09	Incidence also rose in males
Unknown	1.55	1.97	Higher incidence; may reflect coding variability
*Race*			
White	1.31	1.84	Highest incidence in White patients
Black or African American	1.62	2.26	Notable rise across AAP
Asian	1.47	2.19	Substantial increase post-COVID
American Indian / Alaska Native	1.87	2.59	Among the highest rates (small sample size caveat)
Native Hawaiian / PI	0.81	1.09	Lower incidence; rising trend post-COVID
Other Race	1.71	2.81	Highest relative increase among races
Unknown	1.68	2.04	Consistent rise; likely includes mixed coding
*Ethnicity*			
Hispanic or Latino	1.33	2.15	Sharp increase post-COVID
Not Hispanic or Latino	1.30	1.89	Broad increase in incidence
Unknown	1.98	2.33	Elevated rates; may reflect underreporting bias


(B) Baseline Characteristics			
Variables	AUD + AAP with Naltrexone (MAUD)	AUD + AAP without Naltrexone (No MAUD)	*p* value
N total	1,871	31,690	NA
Age at Index (mean ± SD)	46.1 ± 12.2	50.8 ± 13.3	< 0.0001
% Female	31%	29%	0.0783
% Hispanic or Latino	14%	9%	< 0.0001
% White	59%	61%	0.0908
% Black or African American	21%	22%	0.0918
% Asian	2%	2%	0.8200
Type 2 Diabetes Mellitus	25%	25%	0.8634
Coexisting anxiety Disorder	66%	44%	< 0.0001
Coexisting mood Disorders	63%	43%	< 0.0001

Propensity Score Matching											
Cohort 1 (*N* = 1871) and cohort 2 (N = 31,690) characteristics before propensity score matching							Cohort 1 (*N* = 1871) and cohort 2 (*N* = 1871) characteristics after propensity score matching				
Cohort		Mean ± SD	Patients	% of Cohort	*p*-Value	Std diff	Cohort	Mean ± SD	Patients	% of Cohort	*p*-Value
*Demographics*							*Demographics*				
1 2	Age at Index	42.7 ± 11.9 46.1 ± 12.9	1,871 31,690	100% 100%	< 0.001	0.271	1 2	Age at Index	42.7 ± 11.9 42.8 ± 11.8	1,871 1,871	100% 100%
1 2	White		1,101 19,271	58.8% 60.8%	0.091	0.040	1 2	White		1,101 1,103	58.8% 59.0%
1 2	Female		583 9,270	31.2% 29.3%	0.078	0.042	1 2	Female		583 580	31.2% 31.0%
1 2	Hispanic or Latino		263 2,749	14.1% 8.7%	< 0.001	0.170	1 2	Hispanic or Latino		263 241	14.1% 12.9%
1 2	Black or African American		388 7,101	20.7% 22.4%	0.092	0.041	1 2	Black or African American		388 396	20.7% 21.2%
1 2	Asian		37 651	2.0% 2.1%	0.820	0.005	1 2	Asian		37 34	2.0% 1.8%
*Diagnosis*							*Diagnosis*				
1 2	Type 2 diabetes mellitus		322 5,648	17.2% 17.8%	0.501	0.016	1 2	Type 2 diabetes mellitus		322 314	17.2% 16.8%
1 2	Coexisting anxiety disorders		1,064 10,391	56.9% 32.8%	< 0.001	0.499	1 2	Coexisting anxiety disorders		1,064 1,076	56.9% 57.5%
1 2	Coexisting mood disorders		1,033 10,345	55.2% 32.6%	< 0.001	0.467	1 2	Coexisting mood disorders		1,033 1,038	55.2% 55.5%

Clinical Outcomes							
Outcomes	Timepoint	OR pre-PSM	95% CI (Pre)	*p*-value	OR post-PSM	95% CI (Post)	*p*-value
Mortality	1 month	0.239	(0.128, 0.447)	< 0.001	0.274	(0.136, 0.554)	< 0.001
	6 months	0.525	(0.382, 0.722)	< 0.001	0.571	(0.384, 0.847)	0.005
	12 months	0.698	(0.546, 0.892)	0.004	0.785	(0.569, 1.084)	0.141
Chronic Pancreatitis	1 month	0.835	(0.702, 0.993)	0.041	0.718	(0.573, 0.899)	0.004
	6 months	1.146	(1.016, 1.292)	0.026	0.936	(0.795, 1.102)	0.429
	12 months	1.162	(1.039, 1.299)	0.009	0.973	(0.835, 1.134)	0.726
Alcoholic Hepatitis	1 month	0.670	(0.537, 0.836)	< 0.001	0.561	(0.427, 0.738)	< 0.001
	6 months	1.232	(1.068, 1.421)	0.004	0.976	(0.803, 1.185)	0.804
	12 months	1.389	(1.224, 1.578)	< 0.001	0.992	(0.834, 1.180)	0.930
Cirrhosis	1 month	0.672	(0.535, 0.843)	0.001	0.761	(0.565, 1.024)	0.071
	6 months	0.892	(0.757, 1.050)	0.170	0.915	(0.733, 1.141)	0.429
	12 months	0.975	(0.842, 1.130)	0.739	0.974	(0.796, 1.191)	0.797
Hospital Readmission	1 month	0.332	(0.300, 0.367)	< 0.001	0.304	(0.266, 0.348)	< 0.001
	6 months	0.519	(0.473, 0.570)	< 0.001	0.439	(0.384, 0.502)	< 0.001
	12 months	0.566	(0.515, 0.622)	< 0.001	0.488	(0.425, 0.560)	< 0.001
Emergency Department (ED) Visit	1 month	1.227	(1.074, 1.403)	0.003	1.109	(0.921, 1.335)	0.276
	6 months	1.512	(1.374, 1.664)	< 0.001	1.368	(1.197, 1.564)	0.001
	12 months	1.449	(1.320, 1.591)	< 0.001	1.246	(1.095, 1.417)	0.001

### Baseline Characteristics

**Fig. 2:**
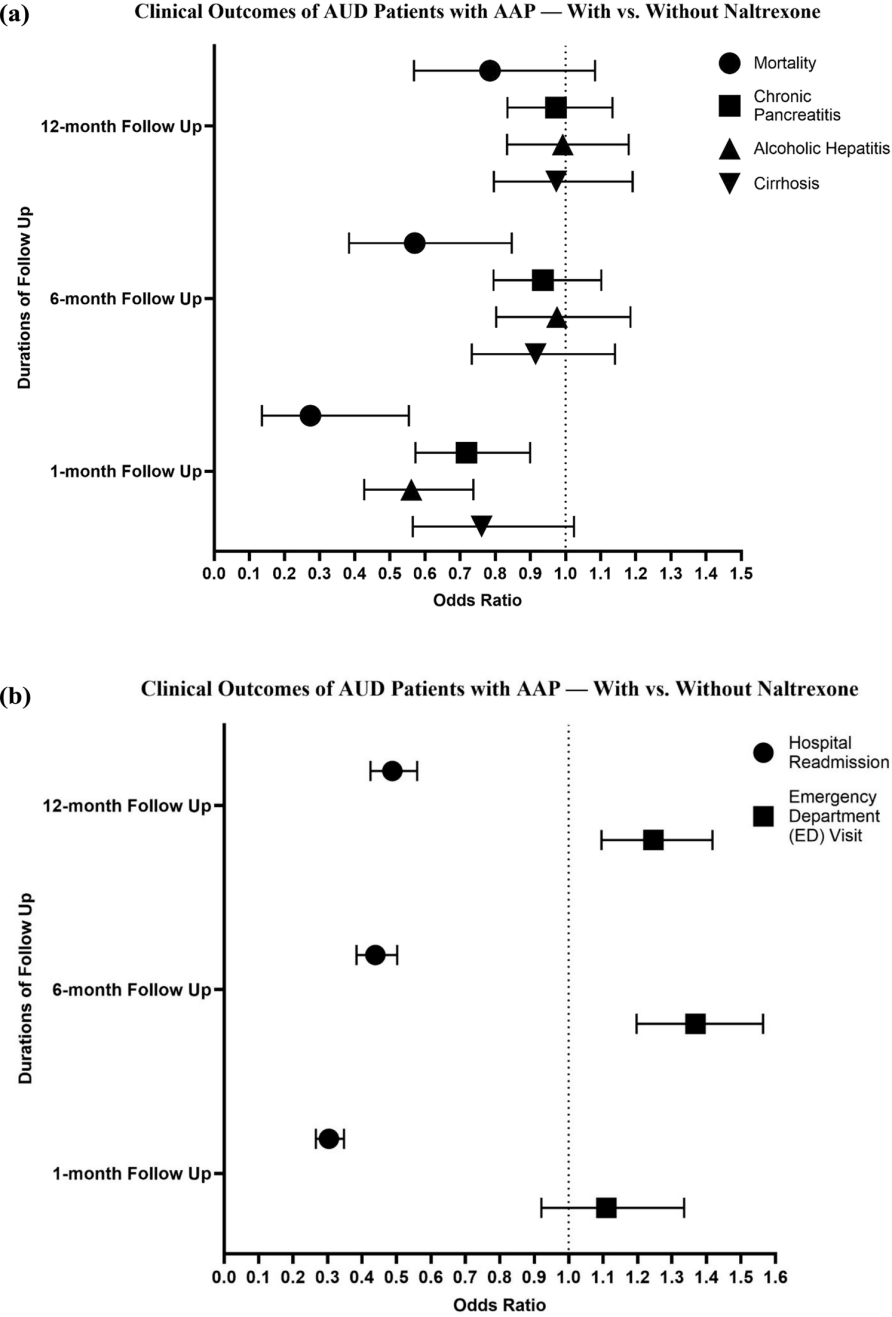
Forest Plot of odds ratios (post-PSM) of clinical outcomes of AUD Patients with AAP — With vs. Without Naltrexone. **A** Clinical outcomes of mortality, chronic pancreatitis, alcoholic hepatitis, and cirrhosis; **B** Clinical outcomes of hospital readmission and emergency department visit

Of the 33,561 AUD patients who developed AAP, only 1,871 (5.6%) were treated with naltrexone around the time of the pancreatitis episode. Table 1B presents the baseline characteristics of AAP patients with vs. without naltrexone. Briefly, patients who received naltrexone tended to be younger (mean age 46.1 vs. 50.8 years, *p* < 0.0001 before matching) and were more likely to be Hispanic (14% vs. 9%, *p* < 0.0001). They also had significantly higher prevalence of coexisting psychiatric disorders: anxiety disorders in 66% vs. 44%, and mood disorders in 63% vs. 43% (both *p* < 0.0001). There were no significant differences in sex distribution (31% female in both) or in baseline rates of type 2 diabetes (25% each) (Fig. 2).

### Clinical Outcomes (Naltrexone vs. No Naltrexone)

We achieved successful 1:1 PSM of the naltrexone and non-naltrexone groups (*N* = 1871 in each after PSM), balancing all the measured covariates (all post-match standardized mean differences < 0.1). In the propensity-matched analysis, naltrexone use was associated with improved short-term outcomes in AUD patients with AAP.

#### Mortality

Patients who received naltrexone had significantly lower all-cause mortality in the early follow-up period. At 1 month, mortality in the naltrexone group was 73% lower than in the no-naltrexone group (OR 0.274, *p* < 0.001). This mortality benefit persisted at 6 months (OR 0.571, *p* = 0.005). By 12 months, the cumulative mortality difference had attenuated and was not statistically significant (OR 0.785, *p* = 0.14).

#### Alcohol-Related Liver Outcomes

Naltrexone-treated patients had lower odds of developing alcoholic hepatitis during follow-up, though this was significant only in the short-term. At 1 month, the incidence of new alcoholic hepatitis diagnoses was 44% lower in the naltrexone group vs. controls (OR 0.561, *p* < 0.001). This difference was no longer significant at 6 or 12 months. There was no significant difference in new diagnoses of cirrhosis between groups at any time point (*p* > 0.1 at 6 and 12 months; rates were low in both groups given that we excluded advanced liver disease at baseline).

#### Chronic Pancreatitis

The naltrexone group showed a modest reduction in progression to chronic pancreatitis. By 1 month, naltrexone was associated with lower odds of a new chronic pancreatitis diagnosis (OR 0.718, *p* = 0.004). However, at 6 and 12 months this difference was not statistically significant.

#### Hospital Readmissions

Notably, hospital readmission rates were substantially lower in the naltrexone group at all assessed intervals. Within 1 month, only 5% of naltrexone patients had been readmitted, compared to 15% of those without naltrexone (OR 0.304, 95% CI 0.266–0.348; *p* < 0.001). This 70% risk reduction in early readmissions persisted in relative magnitude: by 6 months, the naltrexone group’s odds of any readmission were about 56% lower (OR 0.439, *p* < 0.001), and at 12 months they were 51% lower (OR 0.488, *p* < 0.001) than the non-naltrexone group.

#### Emergency Department (ED) Visits

In contrast to the above outcomes, ED visit frequency was higher in the naltrexone group over time. While 1-month ED visit rates were similar, by 6 months the naltrexone group had greater odds of having made an ED visit (OR 1.368, *p* < 0.01). At 12 months, this association persisted (OR 1.246, *p* < 0.01). Numerically, approximately 25% of naltrexone patients had at least one ED visit in the year following AAP, vs. 20% of those not on naltrexone.

## Discussion

In this large multicenter analysis of AUD patients, we found that the incidence of AAP increased significantly during the COVID-19 era, from 1.42% of AUD patients pre-2020 to 1.98% in 2020–2025. The most pronounced relative increases in AAP were observed in young adults, women, and non-white individuals. These subgroup trends suggest that the COVID-19 period saw a broad-based escalation in the burden of AAP, with disproportionately large increases in traditionally “lower-risk” groups (younger adults, women, and non-white individuals) that were perhaps less affected pre-pandemic. This is consistent with national data showing a pandemic-era surge in alcohol-related harms (e.g., a sharp rise in alcohol-related mortality in 2020) [3]. Our findings likely reflect both increased alcohol consumption during periods of lockdown/stress and reduced access to preventive care or AUD treatment among these vulnerable groups. Prior studies have noted similar shifts in pancreatitis etiology; for example, one Italian center reported that the proportion of AP cases attributable to alcohol doubled in 2020 (rising from 6.9 to 12.9% of pancreatitis cases) [8]. Our results underscore this concerning trend of rising AAP incidence in the U.S. during and after the COVID-19 pandemic.

Consistent with prior literature, we observed that only a small minority of eligible patients with AUD and AAP received MAUDs, although studies have consistently shown the benefits of MAUDs [7]. In our cohort, 5.6% of AAP patients were prescribed naltrexone, which is comparable to the generally low uptake of MAUDs nationally (typically < 10% of AUD patients receive any MAUD) [9]. Importantly, however, those AUD patients with AAP who were treated with naltrexone had substantially better short-term outcomes than those who were not. After matching for clinical factors, naltrexone use was associated with significantly lower 1-month and 6-month mortality, as well as markedly fewer hospital readmissions across all time points. These benefits likely stem from improved alcohol abstinence and reduced disease recurrence/severity in patients taking naltrexone. Indeed, high-quality trials have shown that naltrexone therapy decreases the likelihood of returning to heavy drinking, and it is endorsed as a first-line AUD therapy [4]. By helping patients maintain sobriety after the AP episode, naltrexone may mitigate the cycle of relapse that often leads to recurrent pancreatitis or other complications. Our findings align with a recent report in patients with alcohol-related liver disease, where naltrexone prescribing was linked to lower risks of hepatic decompensation and mortality [7]. To our knowledge, this is the first study to demonstrate a similar association in patients with AP. The magnitude of effect we observed (e.g., OR 0.27 for 1-month mortality; OR 0.30 for 1-month readmission) suggests that naltrexone might dramatically attenuate early complications of AAP—perhaps by preventing immediate post-discharge relapse into heavy drinking, which is a common trigger for pancreatitis recurrence.

The observed increase in frequency of ED visits among naltrexone users was somewhat unexpected. Naltrexone is generally well tolerated, although it can cause nausea or other side effects in a subset of patients [10]. One interpretation is that this finding reflects increased healthcare engagement or vigilance: patients on naltrexone (and their providers) might be more proactive in seeking medical evaluation for concerning symptoms, thus leading to more ED visits. Additionally, the naltrexone-treated group had higher baseline rates of psychiatric illness (e.g., anxiety and mood disorders), which could predispose them to utilize emergency services more often (for mental health crises, withdrawal fears, etc.). We cannot determine from our data whether the excess ED visits were directly related to naltrexone side effects (e.g., an intolerable nausea episode prompting an ED trip) or due to these broader behavioral factors. Regardless, this finding underscores the importance of close outpatient follow-up for patients started on AUD medication after AP.

Our study’s limitations warrant discussion. First, it was a retrospective analysis reliant on EHR coding, which may have misclassified some diagnoses or missed instances of alcohol use or milder pancreatitis managed outpatient. Second, only a small subset (5.6%) of AAP patients received naltrexone, which limits the sample size for comparisons and the generalizability of our findings. Those who were prescribed naltrexone were younger and had more psychiatric comorbidities, indicating a selection bias (perhaps reflecting clinicians’ tendency to prescribe AUD medications to more motivated or clinically engaged patients) who may not be fully accounted for even with propensity matching. Third, we lacked detailed information on medication adherence or duration of naltrexone therapy—for example, it was not possible to determine how many patients refilled naltrexone or continued it for months vs. stopped after a brief trial. This could affect outcomes, as sustained AUD treatment would be expected to confer more benefit than a short course. Fourth, although we matched and balanced many covariates, there may be unmeasured confounders influencing our results—such as the severity of AUD (quantity/frequency of drinking prior to AAP), history of relapse, social support, or access to addiction counseling. These factors could impact both the likelihood of receiving naltrexone and the outcomes. Finally, as with any observational study, we can only demonstrate associations, not causation. We advise caution in interpreting the mortality and readmission differences as being caused by naltrexone per se; they could partly reflect the characteristics of patients willing/able to engage in treatment. Prospective studies or trials would be needed to establish causality.

In conclusion, this study highlights two important findings at the intersection of addiction medicine and pancreatology. First, there has been a significant rise in AAP following the COVID-19 pandemic, which appears to have disproportionately affected young adults, women, and minority populations. This calls for public health attention to alcohol use patterns during times of societal stress and possibly for targeted preventive efforts in those groups. Second, among patients with AAP and AUD, the use of naltrexone was associated with improved clinical outcomes—notably, reduced short-term mortality and fewer hospital readmissions—without any observed major safety concerns. These data, in line with evidence from other alcohol-related diseases, support the broader adoption of medications for AUD in patients recovering from AAP. Proactive screening for AUD and initiation of MAUDs (such as naltrexone) during the index hospitalization for AAP could be a high-yield strategy to break the cycle of recurrent illness. Ultimately, integrating AUD treatment into the care of AAP patients may reduce complications, prevent pancreatitis recurrence, and improve survival and quality of life for this high-risk population.

## Supplementary Information

Below is the link to the electronic supplementary material.

Supplementary file 1 (DOCX 33 KB)

Supplementary file 2 (DOCX 22 KB)

## Data Availability

All data supporting the findings of this study are available within the paper and its supplemental material.
