# Supplementary material for: Trends and Outcomes of Alcoholic Acute Pancreatitis in Patients with Alcohol Use Disorder Treated with Naltrexone in the United States: Before and After the COVID-19 Pandemic
Source: Dig Dis Sci. 2025 Sep 29;71(3):880–8. doi: 10.1007/s10620-025-09411-2 (PMC12982193; doi:10.1007/s10620-025-09411-2)
Supplement: Supplementary file 2 — (DOCX 22 KB) [file 10620_2025_9411_MOESM2_ESM.docx]

**Supplemental methods:**

***Exclusion Criteria***

We excluded patients with non-alcoholic causes of acute pancreatitis to ensure etiologic specificity, including gallstone-related pancreatitis (e.g., cholelithiasis, choledocholithiasis, biliary obstruction), hypertriglyceridemia (serum triglyceride level >1000 mg/dL), pancreatic neoplasm or cystic neoplasms, trauma-related pancreatitis, drug-induced pancreatitis (e.g., valproic acid, azathioprine, and furosemide), and autoimmune pancreatitis. We also excluded preexisting advanced liver disease to isolate the effect of naltrexone and reduce confounding factors, which included decompensated cirrhosis (e.g., ascites, variceal bleeding, hepatic encephalopathy), hepatocellular carcinoma (HCC), any prior liver transplantation, or MELD ≥25. Other confounding substance use disorders, such as opioid use disorder (due to direct pharmacological interaction with naltrexone), methamphetamine, cocaine, or polysubstance abuse (as these confound behavioral and clinical outcomes), were also excluded. Pregnant or postpartum individuals (due to different metabolic and clinical pathways affecting outcomes) were excluded from the study. Any prior/concurrent malignancy, immunosuppressed status (including those with immunocompromised diseases, such as human immunodeficiency virus (HIV) infection, acquired immune deficiency syndrome (AIDS), chronic inflammation including autoimmune diseases, or those who were taking immunosuppressants), or those with prior or concurrent organ transplants, were excluded at the index date (as mortality/readmission risks were confounded).

***Propensity Score Matching (PSM)***

We applied a 1 to 1 propensity score matching using nearest-neighbor greedy algorithms. Each component of the risk factors / confounders of alcoholic acute pancreatitis (AAP), alcohol use disorder (AUD), naltrexone pharmacotherapy, and clinical outcomes, was individually matched for (i.e. Age at Index Event, demographics [age, sex, and sex], comorbid conditions based on Elixhauser/Charlson variables (to control overall disease burden), such as chronic kidney disease (CKD), chronic obstructive pulmonary disease (COPD), congestive heart failure (CHF), hypertension, obesity (BMI ≥30), or tobacco use; liver-specific comorbidities such as prior diagnosis of alcoholic hepatitis or hepatic synthetic function markers (bilirubin, INR, albumin levels); prescription of other MAUD agents (acamprosate, disulfiram, topiramate) and antidepressant or antipsychotic medication use; ICU admission or mechanical ventilation during index AAP hospitalization; severity and chronicity of AUD, including benzodiazepine use (reflect withdrawal management or psychiatric severity); other psychological/psychiatric comorbidities beyond mood/anxiety disorders, such post-traumatic stress disorder (PTSD), personality disorders, schizophrenia or other psychotic disorders, or ADHD (linked to higher substance use relapse rates); chronic pain and opioid use conditions, including chronic pain syndromes (e.g., fibromyalgia, chronic back pain) or long-term opioid prescription (may interact with naltrexone contraindications); COVID-19-specific confounders, such as COVID-19 infection status (before or during index AAP); and other alcohol-related comorbidities, including pancreatic insufficiency or malabsorption, alcohol-related cardiomyopathy, cognitive impairment, or Wernicke-Korsakoff syndrome.

***Data Providers***
The TriNetX USA network is a de-identified, longitudinal data source that includes both inpatient and outpatient electronic health records (EHR) from participating healthcare organizations (HCO) across the United States. These patient-level data were sourced from a global federated health research network with almost real-time updates (typically updated every 2-4 weeks). Network members included academic medical centers, integrated delivery networks, specialty hospitals, and large specialty physician practices. The dataset contained detailed clinical information, including demographics, diagnoses, prescribed medications, laboratory test results, vital signs, and procedures performed for each medical encounter and day of hospital stay within the network.

***Data Privacy and Internal Review Board***

TriNetX is structured to allow patient-level analyses while reporting only population-level data to maintain subject anonymity. TriNetX treats all counts between one to ten as equivalent to further maintain anonymity. Because of the de-identified and aggregated nature of the data in the database at the standard defined in Section §164.514(a) of the HIPAA Privacy Rule, the Metrohealth Medical Center Institutional Review Board has deemed studies using the TriNetX database exempt from requiring IRB approval.
 
***Data Elements***
Data elements are those in the fixed fields of the EHR (i.e., demographics, laboratory results, vitals, diagnoses, procedures, and prescribed medications), data captured via text mining of progress notes and other documents within the patient’s record, and additional data linked at the patient level (e.g., mortality).
 
TriNetX typically receives data from HCOs and other data providers in one of the two ways.

1. TriNetX ingests data directly from an HCO research repository (e.g., i2b2) into the TriNetX environment.
2. An HCO or data provider sends TriNetX data extracts in the form of CSV files

All patient data in the TriNetX network were harmonized with standard terminologies. Diagnoses in the patients’ medical records were coded according to the International Classification of Diseases, Tenth Revision, Clinical Modification (ICD-10-CM) diagnosis codes. Procedures were defined by the Current Procedural Terminology (CPT), Healthcare Common Procedure Coding System (HCPCS), and International Classification of Diseases, Tenth Revision, Procedure Coding System (ICD-10-PCS) procedure codes. Medication orders were defined by or mapped to RxNorm Ingredient, CPT, HCPCS, and ICD-10-PCS medication codes. Laboratory test results were defined using Logical Observation Identifiers Names and Codes (LOINC).
 
The data are then transformed into a proprietary data schema. This transformation process includes an extensive data quality assessment that includes ‘data cleaning,’ which rejects records that do not meet TriNetX quality standards. The data elements in TriNetX include but are not limited to

- **Demographic data** included elements such as age, gender, race, ethnicity, and US Census region. The race data within the TriNetX data model are consistent with the HL7 CDC Version 1 standards, which include **American Indian or Alaska Native, Black or African American, Native Hawaiian or Other Pacific Islander, and White. Race and ethnicity data are sourced directly from the provider HCOs and may be either self-reported or**observed by providers in patients’ EHRs.
- **Encounter data** included elements such as start date, end date, and encounter type (ambulatory, emergency room, inpatient, home health, inpatient non-acute, observation, pre-admission, short stay, and virtual).
- **Diagnosis data** were mapped to the International Classification of Diseases, Tenth Revision, Clinical Modification (ICD-10-CM) diagnosis codes, including diagnosis codes, dates, and whether the diagnosis was indicated as primary, secondary, or unknown. Diagnoses may also be indicated as admitting diagnoses or reasons for visit.
- **Procedure data** were defined by the Current Procedural Terminology (CPT), Healthcare Common Procedure Coding System (HCPCS), and International Classification of Diseases, Tenth Revision, Procedure Coding System (ICD-10-PCS) procedure codes, including code, date, and whether the procedure was indicated as primary, secondary, or unknown.
- **Medication data** were defined by or mapped to RxNorm Ingredient codes, CPT, HCPCS, and ICD-10-PCS medication codes, including code, start date, route of administration, brand, strength, quantity dispensed, and supply days. TriNetX identifies active prescriptions administered by a provider, reported by the pharmacy, or reported by the patient during a medical encounter.
- **Laboratory data** were mapped to Logical Observation Identifiers Names and Codes (LOINC) or TriNetX custom codes, and included code, date recorded, lab results (numeric or positive/negative/unknown), and units of measure.
- **Vital signs** are mapped to LOINC or TriNetX custom codes and include the code, date, value, and units of measure.
- There are also oncology- and genomic-specific data types that cover tumor morphology, cancer staging, oncology treatments, and genetic variants.

Only the data elements listed above are reported as available and appropriate. Any data elements derived by TriNetX or natural language processing are indicated in the data tables.
